# Novel Kinesin Family Member 1A Variants Linked to Atypical Parkinsonism Elicit Altered Neuronal Transactive Response DNA Binding Protein 43 kDa Interactions and Dendritic Atrophy

**DOI:** 10.1016/j.ajpath.2025.05.018

**Published:** 2025-06-19

**Authors:** Houman Homayoun, Michael R. DeChellis-Marks, Julia Kofler, Gabriella Fricklas, Amanda M. Gleixner, Fang-Cheng Yeh, David Lacomis, Charleen T. Chu, Christopher J. Donnelly

**Affiliations:** ∗ Department of Neurology, University of Pittsburgh School of Medicine, Pittsburgh, Pennsylvania; † Department of Neurobiology, LiveLikeLou Center for ALS Research, University of Pittsburgh School of Medicine, Pittsburgh, Pennsylvania; ‡ Center for Neuroscience, University of Pittsburgh, Pittsburgh, Pennsylvania; § Department of Pathology, University of Pittsburgh School of Medicine, Pittsburgh, Pennsylvania; ¶ Department of Neurological Surgery, University of Pittsburgh School of Medicine, Pittsburgh, Pennsylvania

## Abstract

Analysis of induced pluripotent stem cell (iPSC)–derived neurons from the son of a father-son pair with novel familial variants of uncertain significance in kinesin family member 1A (KIF1A) [c.408C>G (p.Asp136Glu); c.3914G>A (p.Arg1305His)] reveal pathologic features of altered transactive response DNA binding protein 43 kDa (TDP-43) localization, interactions, and stunted dendritic arbors. Both patients developed spasticity and parkinsonism in their mid-60s, with the father dying at age 70 years. There was impaired putamenal dopamine uptake with preserved uptake in the caudate nuclei, and decreased anisotropy by tractography in multiple motor pathways. Given shared transcriptional mechanisms of hindbrain and spinal cord developmental patterning among neurons of the motor circuitry, iPSC-derived motor neurons from fibroblasts donated by the son were generated to investigate the impact of KIF1A mutations on TDP-43 subcellular localization, biochemical interactions of endogenous wild type and mutant KIF1A and endogenous TDP-43, and the pathologic impact of these KIF1A variants on dendritic arborization using Sholl analysis. Neuropathologic assessment of the father, who shared the same KIF1A variants, revealed tauopathy and TDP-43 proteinopathy throughout the brainstem. Quantitative imaging of patient iPSC neurons identified TDP-43 mislocalization to the soma and dendritic atrophy. The KIF1A mutant also elicited decreased biochemical interactions of both itself and TDP-43 with a spectrum of known TDP-43–associated proteins. These data suggest that this novel KIF1A mutant mediates altered TDP-43 interactions, stunting of the synaptic architecture, and clinical phenotypes coincident with neurodegenerative movement disorders.

*KIF1A* encodes for kinesin-like protein KIF1A (Q12756), a neuron-specific member of the kinesin-3 family involved in long distance cargo transport.^1^ Dominant and recessive variants in the *KIF1A* gene are associated with various central and peripheral nervous system disease phenotypes, including hereditary spastic paraparesis/paraplegia, cerebellar ataxia, autonomic-sensory neuropathy, and axonal sensorimotor polyneuropathy.^2^^,^^3^

Induced pluripotent stem cells (iPSCs) from a patient with a novel familial *KIF1A* variant, and an unusual phenotype of spasticity and parkinsonism, reveal alterations in transactive response DNA binding protein 43 kDa (TDP-43) biology associated with impaired neuronal arborization. Clinical and advanced imaging technologies were used to characterize the patient in the context of neuropathologic findings in his father. Endogenous expression of this mutant KIF1A alters the intracellular localization of TDP-43 with proteomic analyses supporting a biophysical interaction between KIF1A and TDP-43. The variant also elicits decreased protein interactions of TDP-43 and has pathogenic effects on neuronal arborization.

## Materials and Methods

### Clinical Features of the Disease Model

A 65-year-old man with a family history of progressive supranuclear palsy (PSP) was evaluated for weakness and stiffness that started 2 years earlier in his left leg. He initially noted difficulty with kicking his leg during swimming. After 1 year, he progressed to left leg shaking, then stiffness with hyperreflexia, spasticity, and subtle posturing in his left arm. Electrodiagnostic testing was normal. Brain and spinal cord magnetic resonance imaging was normal, aside from mild lumbar stenosis.

Over the next 6 months, he developed markedly asymmetric mixed rigidity and spasticity, along with left leg clonus, a fixed dystonic posture, irregular action tremor, stimulus-induced myoclonus, and bradykinesia in the left arm. His gait exhibited stiffness, left leg dragging, left arm reduced swing, and *en bloc* turns. There was no cognitive impairment, muscle atrophy, weakness, cerebellar ataxia, extraocular motility deficits, or sensory loss.

**Figure 1:**
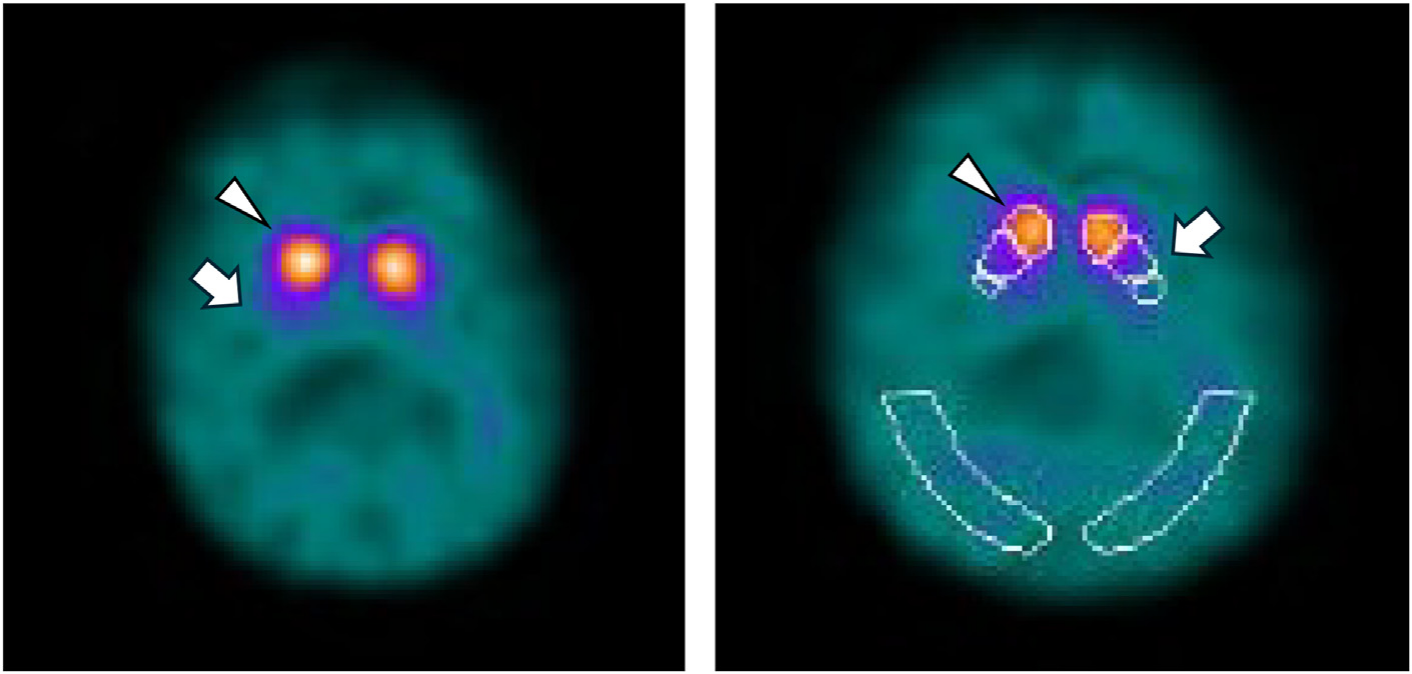
DaTSCAN of the patient. Brain ^123^I-ioflupane imaging (DaTSCAN) in the patient revealed absence of uptake in bilateral putamen (**arrows**) with preserved uptake in the caudate nuclei (**arrowheads**). **Right panel:** From anterior (top) to posterior (bottom), the bilateral white outlined areas delineate the caudate, anterior putamen, posterior putamen, and a control region known to lack dopamine transporter expression.

**Figure 2:**
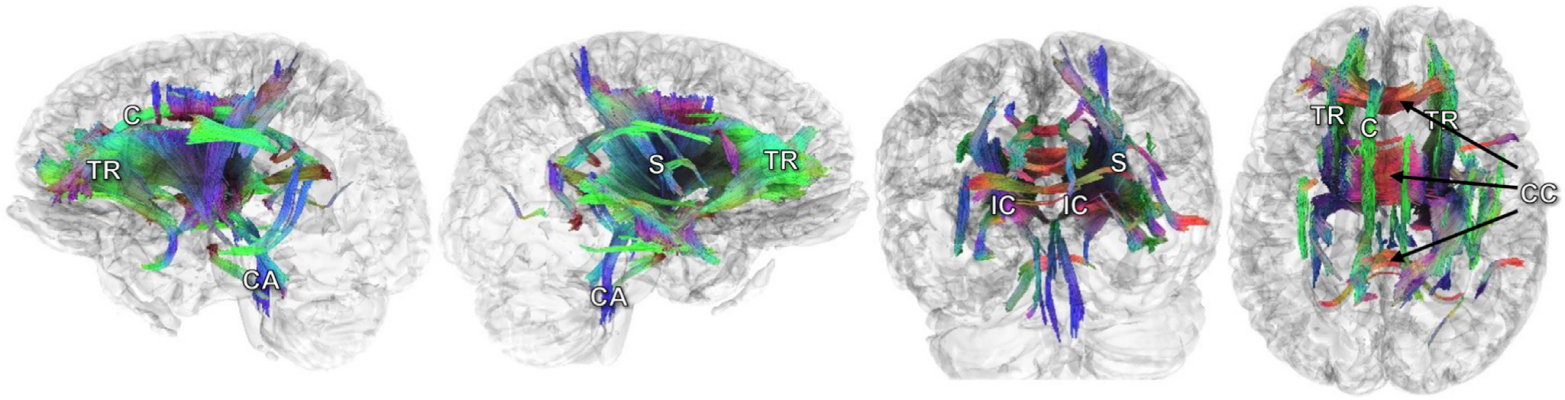
Differential tractography study of the patient. Differential tractography showing pathways with decreases of anisotropy. Differential tractography shows decreased anisotropy in multiple pathways in the internal capsule (IC), right striatum (S), anterior thalamic radiation (TR), corpus callosum (CC), cingulum (C), and tracts close to the cerebral aqueduct (CA). The three-dimensional visualizations are shown from left, right, posterior, and superior views, respectively.

Repeat electrodiagnostic testing was unremarkable. Laboratory studies were normal/negative, including complete metabolic panel, vitamin B12, copper, ceruloplasmin, antibodies for Lyme, human T-lymphotropic virus 1, Whipple disease, and syphilis, paraneoplastic and autoimmune antibody panels, and glutamic acid decarboxylase 65. DaTSCAN (^123^I-ioflupane) revealed a loss of uptake in the bilateral putamen (Figure 1). Differential tractography showed decreased anisotropy, which is related to axonal injury or degeneration,^4^ in multiple pathways in the internal capsule, right striatum, corpus callosum, cingulum, and periaqueductal region. There was degradation of cerebellar connections to the brainstem but no changes in corticospinal tracts (Figure 2).

Given the mixed clinical features, atypical primary lateral sclerosis and atypical parkinsonism were initially considered diagnostically. There was no benefit from levodopa at 600 to 900 mg/d, but he experienced partial improvement in bradykinesia, gait, and dystonia after the dose was increased to 1200 mg/d. Shortly thereafter, he developed levodopa-induced dyskinesias. His spasticity remained unchanged, and he developed new mild weakness on the right side without atrophy or fasciculations. At a 2-year follow-up, a levodopa on-off challenge revealed a 31% improvement on the United Parkinson Disease Rating Scale part III motor scale with medication, with no cognitive impairment by neuropsychological testing.

The family history of paternal PSP was revisited. His father had a rapidly progressive and medication-refractory course characterized by mid-60s onset, axial more than appendicular parkinsonism, early postural instability, and supranuclear gaze palsy with preferential vertical gaze impairment. He developed frequent falls, progressive immobility, bulbar and cognitive impairment, respiratory stridor, and died from respiratory failure at age 70 years. Brain computed tomography scan revealed extensive cerebellar and pontine atrophy and ex vacuo dilatation of all ventricles. His brain autopsy had been initially interpreted as consistent with PSP, but later re-examination identified additional pathologic features described below.

Patient genetic testing revealed two variants of uncertain significance (VUSs) in *KIF1A*, c.3914G>A (p.Arg1305His) and c.408C>G (p.Asp136Glu), with negative tests for *C9orf72*, *MAPT, TARDB, VCP*, and *GRN* mutations. Examination of the father's post-mortem tissue revealed the same VUSs in *KIF1A*. Moreover, pathologic TDP-43 inclusions were noted in multiple regions of the ventral brainstem that coordinate motor output, including the basis pontis, oculomotor nucleus, and olivocerebellar pathway. Mutations in *KIF1A* are associated with neurodevelopmental and neurodegenerative disorders through various mechanisms.^5^, ^6^, ^7^ The atypical clinical phenotype and the father's pathologic findings supported the hypotheses that the identified *KIF1A* VUSs could be pathogenic and linked to TDP-43 proteinopathy.

Given the unusual pattern of distribution of TDP-43 in the father, the impact of KIF1A mutations on TDP-43 localization was studied using iPSC-derived motor neurons from the son. Transcriptional mechanisms of hindbrain and spinal cord developmental patterning are shared among motor neurons and associated interneurons of the motor circuitry.^8^^,^^9^ Affinity-purification mass spectrometry (AP-MS) was used to evaluate the proteomic relationships between wild-type and mutant KIF1A and TDP-43. Sholl analysis was used to study the impact of these mutations on neuronal morphology.

### Pathology

The patient's father died at age 70 years from laryngeal dysfunction and bilateral bronchopneumonia. His brain tissue sections were examined using hematoxylin and eosin and Bielschowsky silver stains. The original tissue blocks were retrieved, and additional immunohistochemical stains were performed for phosphorylated tau (PHF1; 1:1000; pH 6.0; citric acid pretreatment; courtesy of Peter Davies, formerly of Albert Einstein College of Medicine, New York, NY), phosphorylated TDP43 (1D3; 1:500; pH 6.0; citric acid pretreatment; BioLegend, San Diego, CA), and α-synuclein (LB 509; 1:500; protease pretreatment; Santa Cruz Biotechnology, Dallas, TX).

### Tractography

Images were obtained from a SIEMENS Healthineers Prisma scanner (Erlangen, Germany) using a diffusion sequence with TE (echo time) of 99.2 milliseconds and TR (repetition time) of 2490 milliseconds. A diffusion spectrum imaging scheme was used for acquisition, taking 257 diffusion samplings and a maximum b-value of 4000 seconds/mm^2^. The in-plane resolution was 2 mm, and slice thickness was 2 mm. To correct for susceptibility artifact, reversed phase-encoding b0 was estimated using TOPUP from the Tiny FSL package (*http://github.com/frankyeh/TinyFSL*, last accessed March 6, 2023), a recompiled version of FSL TOPUP (Oxford Centre for Functional MRI of the Brain, Oxford, UK) with multithread support. The correction was performed using the integrated interface in DSI Studio (Chen release, developed by F.-C.Y.). The restricted diffusion was quantified using restricted diffusion imaging^10^; generalized q-sampling imaging^11^ was used to reconstruct diffusion data with a diffusion sampling length ratio of 1.25. The tensor metrics were calculated using diffusion-weighted imaging with b-values <1750 seconds/mm^2^.

Differential tractography^12^ was used to map pathways with quantitative anisotropy^4^ decreases >20% in comparison with a normal cohort of 21 individuals (mean ± SD age, 55.7 ± 16.1 years; range, 27 to 74 years; 10 females and 11 males), receiving the identical scan protocol. For comparison with the patient, the anisotropy of the cohort was adjusted on the basis of the sex and age to match the patient.

In differential fiber tracking, the anisotropy threshold, angular threshold, and step size were randomly selected from Otsu threshold, 15 to 90 degrees, and 0.5 to 1.5 voxels, respectively. Tracks with a length <30 or >300 mm were discarded, and a total of 1,000,000 seeds were placed. Topology-informed pruning was applied to the tractography with two iterations to remove false connections.^13^

### Cell Culture

Forearm skin biopsy collected from the KIF1A^D136E, R1305H^ patient was plated in fibroblast growth media (minimal essential medium; Gibco, Evansville, IN; catalog number 11095-080) supplemented with 10% fetal bovine serum (Thermo Fisher, Waltham, MA; catalog number 16000044), 1% non-essential amino acids (Gibco; catalog number 11140-050), and 1% sodium pyruvate (Gibco; catalog number 11360-070). Within 30 days, outgrowing fibroblasts were isolated by trypsinization (Gibco; catalog number 25200056) and transferred to new dish with fibroblast growth media.

Patient-derived iPSCs were generated by reprogramming fibroblasts with ReproRNA-OKSGM kit (STEMCELL Technologies, Vancouver, BC, Canada; catalog number 05930), followed by quality assessment involving immunofluorescence labeling for pluripotency markers, STEMdiff Trilineage Differentiation (STEMCELL Technologies; catalog number 05230), real-time quantitative PCR analysis for germ layer gene expression, and karyotyping for chromosomal abnormalities (WiCell Research Institute, Madison, WI).

The iPSCs were maintained in mTeSR Plus medium (STEMCELL Technologies) on Matrigel (Corning, Corning, NY; 354277). Cultures were routinely monitored for spontaneous differentiation, growth rates, and cellular morphology.

Motor neuron differentiation was conducted as previously described.^14^^,^^15^ Cells were plated at a density of 210,000 cells per cm^2^ in mTeSR Plus medium and 10 μmol/L Y-27632 [Rho-associated coiled-coil containing protein kinase (ROCK) inhibitor; STEMCELL Technologies] with differentiation initiated at 90% confluency. Differentiation occurred through two stages that included daily media changes. N2B27 base media stage 1 is delivered for 6 days and stage 2 is given for 8 days. Cells are then dissociated and plated in Neurobasal medium (Gibco). Cells were matured for a minimum of 14 days before characterization.

### Immunocytochemistry

iPSC-derived motor neurons from patient and controls (Supplemental Table S1) were washed three times in phosphate-buffered saline (PBS) and fixed on glass coverslips (Electron Microscopy Sciences, Hatfield, PA; catalog number 72196-S) at 28 days *in vitro* with 4% paraformaldehyde (Electron Microscopy Sciences; catalog number 15714-S). Following three sequential PBS washes, cells were permeabilized with 0.3% Triton X-100 in 1× PBS and then blocked in 5% normal donkey serum in PBS with 0.3% Triton X-100. Neurons were stained overnight at 4°C in the indicated primary antibody in blocking buffer (Table 1). Secondary antibodies were incubated for 1 hour at room temperature. Coverslips were mounted to glass slides (VWR, Radnor, PA; catalog number 16005-106) using ProLong Glass Antifade Mountant with NucBlue Stain (Thermo Scientific; catalog number P36891) and cured for 48 hours before imaging.

**Table 1:** Primary Antibodies

Primary antibody	Vendor	Catalog no.
KIF1A	Abcam, Cambridge, MA	Ab180153
MAP2	PhosphoSolutions, Davis, CA	1099-MAP2
SSEA4	Developmental Studies Hybridoma Bank, Iowa City, IA	MC-813-70
TDP-43	Proteintech, Rosemont, IL	10782-2
TRA-1-81	StemCell Technologies, Vancouver, BC, Canada	60065
OCT4a	Cell Signaling Technology, Danvers, MA	2480

### RNA Extraction and Real-Time Quantitative PCR

Samples were washed with PBS before RNA extraction using miRNeasy Mini Kit (Qiagen, Hilden, Germany; catalog number 217004). cDNA was then synthesized from equal amounts of RNA using iScript Select cDNA Synthesis Kit (catalog number 170-8897; BioRad, Hercules, CA). The real-time quantitative qPCRs [SsoAdvanced Universal SYBR Green Supermix (BioRad; catalog number 1725272) and 10 μmol/L primers] were run using the BioRad CFX96 Real-Time System in triplicate technical replicates using primers (Integrated DNA Technologies, Coralville, IA) shown in Table 2 for the following genes: SRY-box transcription factor 1 (SOX1), paired box 6 (PAX6), SRY-box transcription factor 17 (SOX17), C-X-C motif chemokine receptor 4 (CXCR4), forkhead box protein F1 (FOXF1), and heart and neural crest derivatives expressed 1 (HAND1). Expression of germ layer genes was compared between differentiated and undifferentiated cells using comparative cycle threshold (C_T_) analysis.

**Table 2:** Trilineage Differentiation Germ Layer Validation Primers

Germ layer	Transcript	Forward primer sequence	Reverse primer sequence
Ectoderm	SOX1	5′-AATACTGGAGACGAACGCCG-3′	5′-CCCTCGAGCAAAGAAAACGC-3′
	PAX6	5′-AGCCCAGTATAAGCGGGAGT-3′	5′-TGTTTATTGATGACACGCTTGGT-3′
Endoderm	SOX17	5′-CCGCACGGAATTTGAACAGT-3′	5′-AATATACCGCGGAGCTGGC-3′
	CXCR4	5′-TTCTTAACTGGCATTGTGGGC-3′	5′-CCCAGAAGGGAAGCGTGAT-3′
Mesoderm	FOXF1	5′-TCTCGCTCAACGAGTGCTTC-3′	5′-GTTCATCATGCTGTACATGGGC-3′
	HAND1	5′-GGTTAAACAGGTCTTTGGGCT-3′	5′-CGGGCAAGGCTGAAAATGAG-3′

### Immunoprecipitation

M-270 epoxy beads (approximately 5 mg; Thermo Fisher Scientific; catalog number 14321D) were washed and prepared according to instructions, mixed with buffers C1 and C2, and 25 μg of anti-KIF1A antibody or anti–TDP-43 antibody (Table 1) and roller-incubated overnight at 37°C. For immunoprecipitation, 1 mg of antibody-coupled beads was washed with 1× cell lysis buffer and separated for immunoprecipitation. Approximately 1 mg of protein from iPSC-neuron lysate was applied to the antibody-coupled beads and incubated for 12 to 16 hours at 4°C. Unbound fractions were collected and stored. Beads were washed again with 1× cell lysis buffer and transferred to a clean tube. Protein was eluted off using 1× Laemmelli buffer with 1% β-mercaptoethanol (Bio-Rad; catalog number 1610710) at 100°C for 10 minutes.

### Sample Preparation for Mass Spectrometry

Cell homogenates were thawed at room temperature, vortexed for 10 minutes, bath sonicated for 5 minutes, and centrifuged at 13,000 × *g* for 10 minutes at room temperature. Supernatants were isolated for protein quantification by a micro–bicinchoninic acid assay (Thermo Fisher Scientific; catalog number 23235). Protein digestion was performed on 10 μg of protein from each sample according to the S-trap (Protifi) protocol. Samples were subject to reduction with 20 mmol/L dithiothreitol, heated to 95°C for 10 minutes, cooled to room temperature, and alkylated by incubation with 40 mmol/L iodoacetamide in darkness for 30 minutes at room temperature. Samples were centrifuged at 13,000 × *g* for 8 minutes, acidified with 12% phosphoric acid at 1:10 concentration, and diluted sixfold in binding buffer containing 90% methanol and 100 mmol/L triethylammonium bicarbonate (TEAB) at pH 7.1. Samples were dispensed onto S-trap columns, which were washed with binding buffer and centrifuged at 4000 × *g* for 1 minute. Columns were washed with binding buffer, centrifuged at 4000 × *g* for 1 minute, and transferred to clean tubes for incubation at 47°C in 50 mmol/L TEAB for trypsin digestion at 1:10 enzyme/substrate. Samples were eluted with a series of solutions, including 50 mmol/L TEAB, 0.2% formic acid, and 50% acetonitrile with 0.2% formic acid, and underwent centrifugation at 1000 × *g* for 1 minute after each eluent was added, then dried in a speed vacuum centrifuge and resuspended in a solution of 3% acetonitrile and 0.1% trifluoroacetic acid and desalted using Pierce Peptide Desalting Spin Columns (Thermo Fisher Scientific; catalog number 89851). Eluants were dried in a speed vacuum centrifuge and resuspended in a solution of 3% acetonitrile and 0.1% formic acid to a final concentration of 0.5 μg/μL.

### Mass Spectrometry

Mass spectrometry (MS) analysis was conducted on a Thermo Fisher QE-HFX coupled to an Easy-nLC 1200. Approximately 1 μg of each sample was loaded onto an EASY-Spray PepMap RSLC C18 column (2 μm; 100 A; 75 μm × 50 cm) and eluted at 300 nL/minute over a 60-minute gradient. MS1 spectra were collected at 60,000 resolution with a full scan range of 350 to 1400 m/z, a maximum injection time of 50 milliseconds, and the automatic gain control set to 3e6. The precursor selection window was 1.4 m/z, and fragmentation was performed with high-energy collision dissociation at 28% nominal collision energy. MS2 were collected with a resolution of 30,000, a maximum injection time of 50 milliseconds, and the automatic gain control set to 1e5, and the dynamic exclusion time set to 90s. Initial MS data analysis was performed using Proteome Discoverer version 3.2 (Thermo Fisher Scientific). MS/MS spectra were searched against the human SwissProt database using Sequest HT (missed cleavages = 2; minimum peptide length = 6; precursor mass tolerance = 10 ppm; dynamic modifications: oxidation/+15.995 Da, acetyl/+42.011 Da, met-loss/–131.040 Da, met-loss + acetyl/–83.030 Da; static modifications: carbamidomethyl/+57.021 Da). Peptide spectral matches were filtered using Percolator with a normalized correlation score (deltaCN) = 0.05 and false discovery rate of 1%. Peptide correlation analysis was performed on peptides as previously described.^16^ The order of sample injection was blinded. Samples were excluded if summed and average peptide intensities were significantly lower than all other samples.

### Plasmids

The lentiviral hSyn::iRFP670 plasmid was generated using the Gibson Assembly method. The human synapsin I promoter (hSyn) was PCR amplified from pLV-hSyn-RFP (Addgene, Watertown, MA; catalog number 22909). The fragment was then inserted into piRFP670-N1 (Addgene; 45457) at AflIII and HindIII restriction enzyme sites to produce base vector hSyn-iRFP670. The hSyn::iRFP fragment was PCR amplified from this base vector and transferred into lentiviral backbone (Addgene; 36084) at BsrGI and NdeI restriction enzyme sites. Primers are in Table 3. Newly generated plasmids were validated by Sanger sequencing (Genewiz). hSyn::iRFP670 plasmid was then packaged into lentiviral particles (OriGene, Rockville, MD; catalog number TR30037).

**Table 3:** Cloning Primers

Precursor	Forward primer sequence	Reverse primer sequence
pLV-hSyn-RFP	5′-GCCTTTTGCTGGCCTTTTGCTCAAGTGCAAGTGGGTTTTAGGACC-3′	5′-GCGTCGACTGCAGAATTCGACTGCGCTCTCAGGCACGA-3’
hSyn-iRFP670	5′-GGTTGATTGTCGACTTACTTTTAGCGTTGGTGGTGGGC-3’	5′-GCAGTACATCAAGTGTATCAAGTGCAAGTGGGTTTTAGGAC-3’

### Confocal Imaging

Confocal imaging was performed using a Nikon A1R confocal with a 60× Apo 1.4 numerical aperture oil immersion objective. Nikon type F immersion oil (catalog number MXA22168; Minato City, Tokyo, Japan) was used as a median between the coverslip and objective. Images were taken at 1024 resolution, with two-times line averaging, using Nikon Elements Nyquist function. Laser power and gain for all lasers (405-, 488-, and 647-nm emission) retained identical settings within individual differentiation experiments as a control in quantitative image analysis.

### Quantitative Image Analysis

Quantitative image analysis was performed using Nikon Elements Software version 5.30. Images received no postacquisition alterations (eg, deconvolution) before quantitative analysis. Maximum intensity projects were generated from Z-stack confocal images before quantitative analysis. The mean intensity fluorescence of TDP-43 was measured manually by tracing the contours of the nucleus (via 405-nm channel) and cytoplasm of the soma (via 647-nm channel). Mean intensity fluorescence of TDP-43 was derived from nucleus and cytoplasm as separate, paired compartments. Automated deconvolution was performed on representative images.

### Neuron Tracing and Sholl Analysis

iPSC-derived motor neurons (28 days *in vitro*) were transduced with a hSyn::iRFP670 lentivirus at 5 multiplicity of infection and fixed in 4% paraformaldehyde. Imaging was performed using a 40× UPlanSApo 1.35 numerical aperture oil-immersion objective on an Olympus IX83 microscope (Shinjuku City, Tokyo, Japan) fitted with a DP80 camera. Dendrite analysis was performed as previously described,^17^ using NIH ImageJ version 1.53j (NIH, Bethesda, MD; *https://imagej.net/ij*) supplemented with the SNT plugin.^18^^,^^19^ Sholl sample size was determined from previous experience. Images were renamed and randomized using the random number generator function of Microsoft Excel (Redmond, WA). Sholl analysis was performed by an individual (G.F.) blinded to the identity of the lines, using a designated center point of the soma with a continuous radius step size of 1 μm. The Sholl area under the curve was calculated as *|a∫b f(x)dx|,* where *f*(*x*) equals the number of intersections at a specific radius using the whole neuron from a = 0 to b = 321 μm.

### Statistical Analysis

Statistical analysis was performed using GraphPad Prism Software version 10.1.1 (GraphPad Software, San Diego, CA); *P* < 0.05 was deemed significant. Unpaired two-way *t*-tests were used when comparing two groups. One-way analysis of variance was used to determine statistical significance when comparing three groups. For Sholl analysis, a repeated-measures two-way analysis of variance with Tukey multiple comparison test was used. No individual data points were excluded. Adjusted *P* values and two-way analysis of variance were used to measure significance in peptide correlation analysis.^16^

## Results

### Father's Pathology

**Figure 3:**
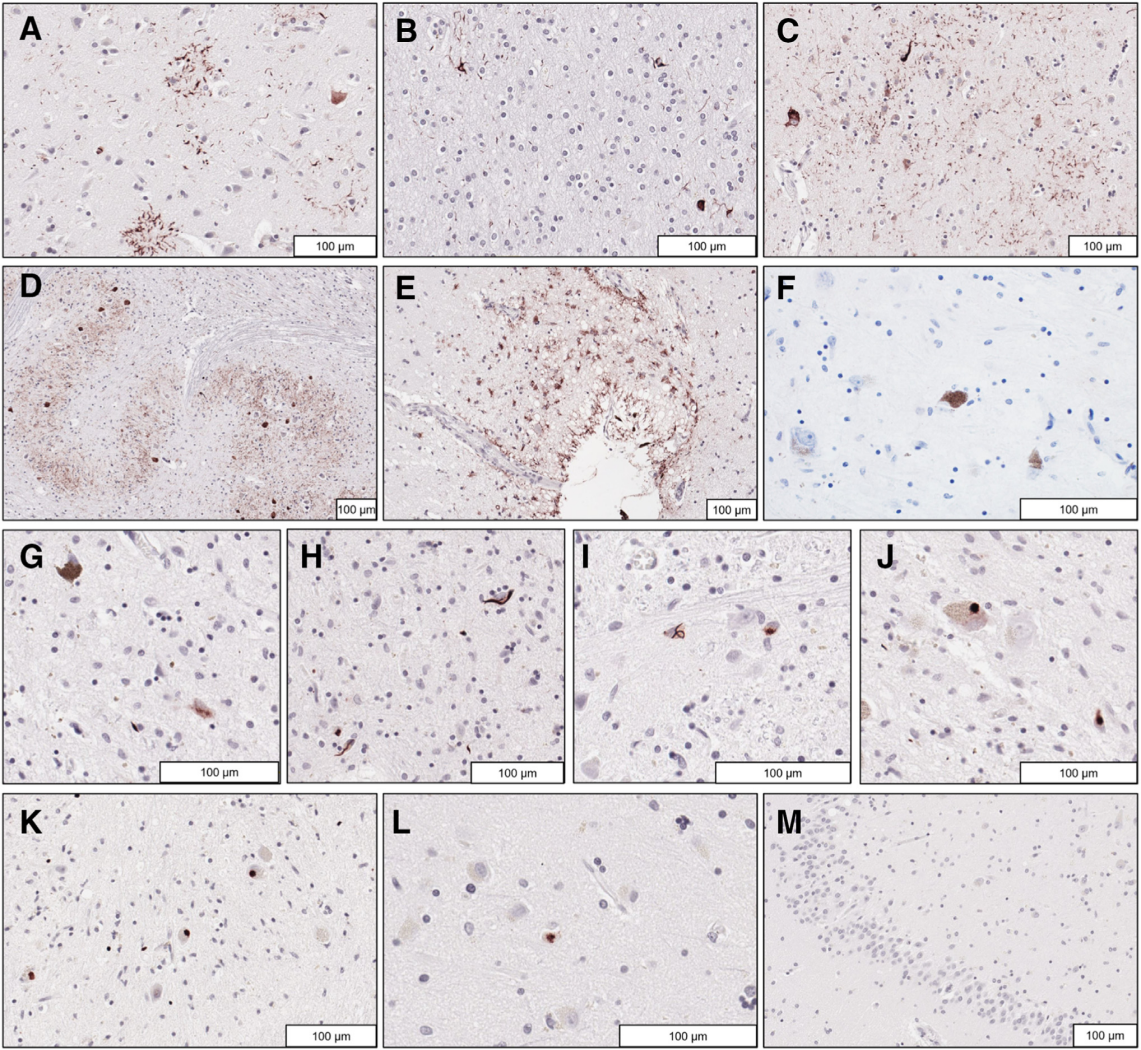
Neuropathologic findings in postmortem examination. **A**–**E:** Phosphorylated tau immunohistochemistry revealed frequent astrocytic plaques in the cortex (**A**), scattered oligodendroglial coiled bodies and threads in the white matter (**B**), tangles, threads, and coiled bodies in the striatum (**C**), numerous tangles and threads in the inferior medulla (**D**), and aging-related thorn-shaped astrocytes in focal subpial distributions (**E**). **F:** α-Synuclein stains were negative for Lewy bodies or Lewy neurites in the substantia nigra. **G**–**K:** Phosphorylated TDP43 immunohistochemistry revealed neuronal and glial cytoplasmic inclusions in the substantia nigra (**G**), tectum (**H**), basis pontis (**I**), oculomotor nucleus (**J**), and inferior olive (**K**). **L** and **M:** Only rare phosphorylated TDP43 neuronal cytoplasmic inclusions were detected in the amygdala (**L**), and none was present in the hippocampus (**M**). Scale bar = 100 μm (**A**–**M**).

Phosphorylated tau immunostaining demonstrated the widespread presence of pretangles, tangles, neuropil threads, astrocytic plaques (Figure 3A), and oligodendroglial coiled bodies (Figure 3B), variably involving cortical, subcortical (Figure 3C), cerebellar, and brainstem gray and white matter (Figure 3D), admixed with aging-related astrocytic tau pathology (Figure 3E). Given the absence of tufted astrocytes and the presence of astrocytic plaques, the tau pathology pattern is more consistent with a diagnosis of corticobasal degeneration (CBD) rather than the earlier pathologic diagnosis of PSP, although the cortical involvement was milder than in typical CBD cases. No neuronal or glial α-synuclein pathology was identified (Figure 3F). Occasional diffuse plaques were focally present on Aβ stains of the neocortex, but negative in other brain regions. No neuritic plaques were identified on Bielschowsky silver-stained sections. However, phosphorylated TDP43 pathology in the form of neuronal cytoplasmic inclusions, a few threads, and a few glial inclusions were observed in the midbrain (Figure 3G), tectum (Figure 3H), basis pontis (Figure 3I), oculomotor (Figure 3J), and inferior olivary nuclei (Figure 3K), with occasional dystrophic neurites in the hypoglossal nucleus and anterior horn motor neurons of the spinal cord. TDP43 pathology was largely restricted to the brainstem with rare amygdala inclusions (Figure 3L) but no involvement of cortical structures, basal ganglia, or hippocampus (Figure 3M). This TDP-43 distribution is unusual and does not fit the criteria for amyotrophic lateral sclerosis, frontotemporal lobar degeneration with TDP43 pathology, or limbic-predominant age-related TDP43 encephalopathy.

### KIF1A Mutant iPSC Line Generation

The KIF1A^D136E,R1305H^ patient provided a forearm skin biopsy. Skin fibroblasts were reprogrammed into iPSC using nonintegrating RNA reprogramming vectors and karyotyped at passage 4 at a band resolution of 375 to 425 with no chromosomal abnormalities detected (Supplemental Figure S1A). KIF1A^D136^^E^^,R1305H^ iPSC lines expressed stage-specific embryonic antigen-1 (SSEA1), tumor-related antigen-1-81 (TRA-1-81), and octamer-binding transcription factor 4A (OCT4a) markers of pluripotency (Supplemental Figure S1B). Capacity for differentiation into three germ layers (ectoderm, endoderm, and mesoderm) was confirmed by trilineage differentiation and quantitative RT-PCR (Supplemental Figure S1C). KIF1A^D136^^E^^,R1305H^ iPSC lines retained D136E and R1350H mutations following reprogramming (Supplemental Figure S1D).

### TDP-43 Is Mislocalized in KIF1A Mutant iPSC Neurons

**Figure 4:**
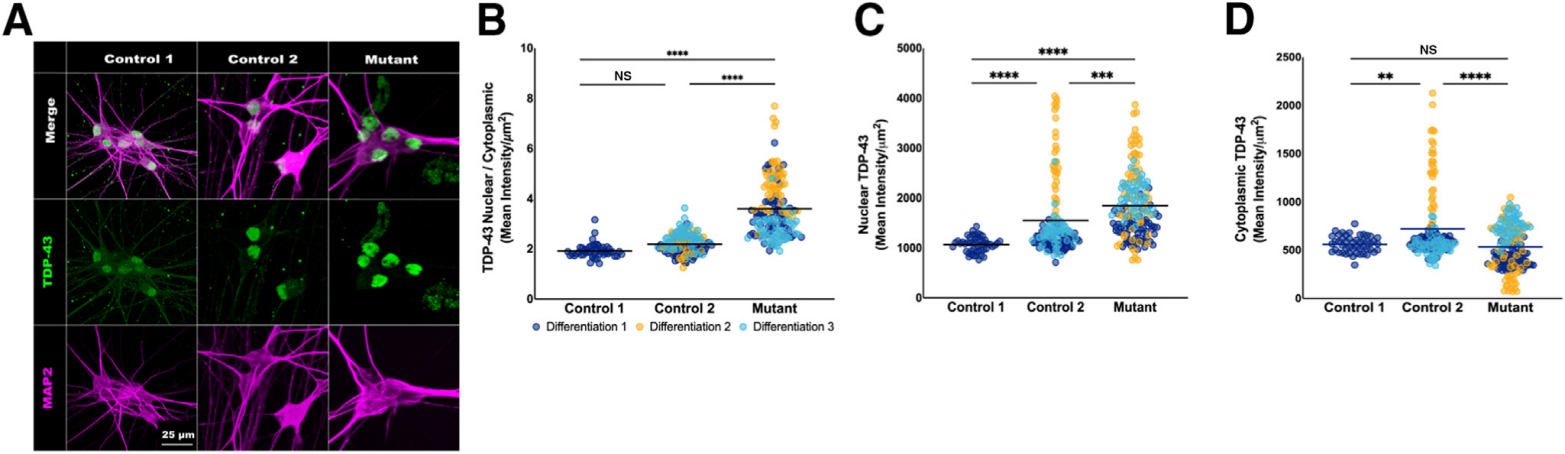
The TDP-43 nuclear/cytosolic ratio is perturbed in KIF1A^D136E, R1305H^ induced pluripotent stem cell (iPSC) motor neurons. Nuclear and cytoplasmic TDP-43 levels (mean intensity fluorescence per μm^2^) were compared in iPSC-derived motor neurons with genotypes of KIF1A wild type (control 1, control 2) and KIF1A^D136E, R1305H^ (mutant). Neurons were collected and evaluated from three separate differentiations (control 2, *n* = 141 neurons; mutant, *n* = 162 neurons). Neurons from control 1 were collected from one differentiation (*n* = 49 neurons). Statistical analyses were performed using parametric one-way analysis of variance with Tukey multiple comparison test. **A:** Representative images of neurons derived from control 1, control 2, and mutant (left to right). Neurons are immunostained with TDP-43 (green) and MAP2 (magenta). **B:** The nuclear/cytoplasmic TDP-43 ratio is significantly increased in mutant neurons (grand mean = 3.604) compared with control 1 (grand mean = 1.922) and control 2 (grand mean = 2.19). This ratio is comparable between control samples. **C:** The level of nuclear TDP-43 is significantly increased in mutant neurons (grand mean = 1848.0) compared with both control 1 (grand mean = 1069.0) and control 2 (grand mean = 1550.0). Nuclear TDP-43 levels are significantly increased in control 2 compared with control 1. **D:** The cytoplasmic levels of TDP-43 are significantly decreased in mutant neurons (mean = 535.6) compared with control 2 (grand mean = 721.4) but not control 1 (grand mean = 562.1, t = 0.7944, Df = 219). Cytoplasmic TDP-43 levels are significantly decreased in control 1 compared with control 2 (t = 3.053, Df = 188). ∗∗*P* < 0.01, ∗∗∗*P* < 0.001, and ∗∗∗∗*P* < 0.0001. Scale bar = 25 μm (**A**). NS, nonsignificant.

Along with iPSC-derived motor neurons from nonneurologic controls [wild type KIF1A (KIF1A^WT^)] obtained from Cedars-Sinai iPSC Core (Supplemental Table S1), the patient iPSC line (KIF1A^D136E,^^R1305H^; hereafter KIF1A^mut^) was matured to 28 days *in vitro* motor neurons, and stained for TDP-43 and microtuble-associated protein 2 (MAP2), a morphologic marker of mature neurons^20^ (Figure 4A). TDP-43 is mislocalized in a spectrum of neurodegenerative diseases.^21^ Calculating the ratio of the mean fluorescence intensity of TDP-43 in the nuclei and soma of KIF1A^mut^ neurons compared with KIF1A^WT^ revealed that the nuclear/cytoplasmic ratio of KIF1A^mut^ neurons was significantly increased compared with two iPSC KIF1A^WT^ controls (Figure 4B). When analyzing each compartment separately, nuclear TDP-43 levels were significantly higher in KIF1A^mut^ neurons compared with two unrelated KIF1A^WT^ controls (Figure 4C), whereas cytoplasmic TDP-43 levels were significantly decreased in KIF1A^mut^ neurons compared with one control line, but not the other (KIF1A^WT^) (Figure 4D). Mutated KIF1A may decrease distal trafficking of TDP-43, leading to enhanced nuclear localization.

### TDP-43 Association with KIF1A^D136E, R1305H^ Is Perturbed

How mutations in KIF1A may cause TDP-43 spatial mislocalization remains unclear. Contemporary evidence suggests that TDP-43 hitchhikes on KIF1A vesicle cargo using annexin 11 as a molecular tether,^22^ representing a direct pathway for fast anterograde axonal transport of TDP-43. AP-MS using KIF1A and TDP-43 antibodies in whole neuron protein lysates was used to determine if KIF1A and TDP-43 interact in neurons.

**Figure 5:**
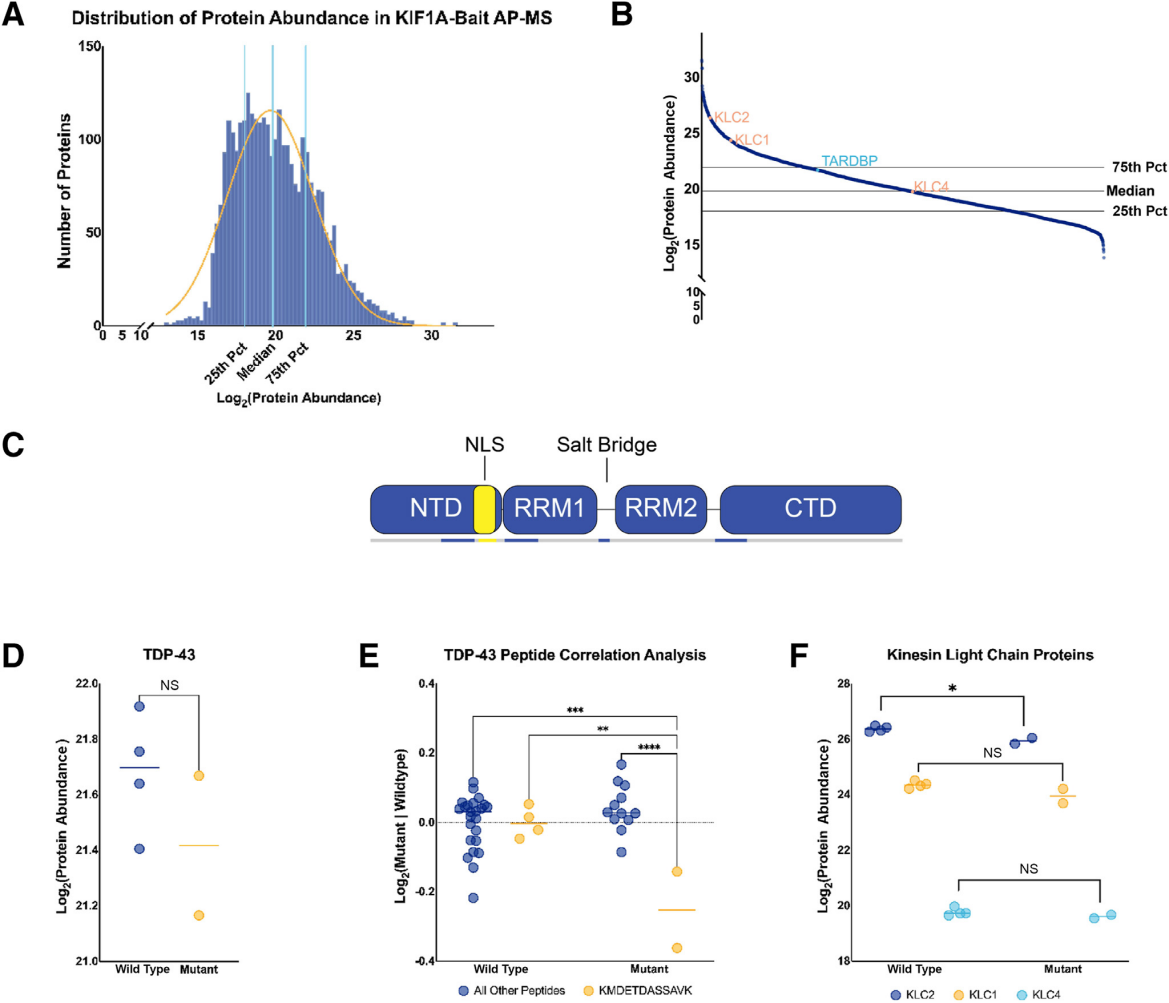
Decreased association between TDP-43 and kinesin light chain protein 2 (KLC2) with mutant KIF1A. Neuronal protein lysates were co-immunoprecipitated from control and mutant neurons using KIF1A as bait. Proteins were identified and quantified using affinity-purification mass spectrometry (AP-MS). **A:** Proteins quantified in the control KIF1A co-immunoprecipitates exhibited a gaussian distribution. **B:** Quantified proteins were sorted by protein abundance level in control neurons with TDP-43/TARDBP (cyan) identified as having protein abundance level near the 75th percentile (Pct). Kinesin light chain proteins, KLC1 and KLC2, locate above the 75th percentile, whereas KLC4 located slightly below the median percentile. **C:** Illustrated representation of TDP-43 and concordant domains. Quantified TDP-43 peptides (blue, gold) are identified throughout the protein until G294. Undetected peptides are noted in gray. The nuclear localization sequence (NLS) in the N-terminal domain of TDP-43 is highlighted in gold, with KMDETDASSAVK denoted in gold text below. **D:** TDP-43 protein levels in KIF1A co-immunoprecipitations are not significantly different between control and mutant (unpaired two-tailed *t*-test, control mean = 21.68, mutant mean = 21.42). **E:** TDP-43 NLS peptide KMDETDASSAVK (AA83-AA96; gold) is significantly decreased in KIF1A co-immunoprecipitation of mutant relative to control, and all other proteins [two-way analysis of variance, F (1, 38) = 16.70; genotype factor, F (1, 38) = 8.521; peptide factor, F (1, 38) = 16.70; control all other peptides versus mutant KMDETDASSAVK, control KMDETDASSAVK versus mutant KMDETDASSAVK, mutant all other peptides versus mutant KMDETDASSAVK). **F:** KLC2 (blue) was significantly decreased in mutant KIF1A co-immunoprecipitates relative to control (control mean = 26.38, mutant mean = 25.94, unpaired two-tailed *t*-test). KLC1 (gold; control mean = 24.36, mutant mean = 23.98) and KLC4 (cyan; control mean = 19.77, mutant mean = 19.60) were not significantly different between groups. ∗*P* < 0.05, ∗∗*P* < 0.01, ∗∗∗*P* < 0.001, and ∗∗∗∗*P* < 0.0001. CTD, C-terminal domain; NS, nonsignificant; NTD, N-terminal domain; RRM, RNA-recognition motif.

Using a KIF1A-bait antibody, two co-immunoprecipitated protein lysates from control 2, control 3, and KIF1A^mut^ iPSC neurons were analyzed. Quality control revealed an abnormally low summed peptide intensity for one replicate of control 1, and this replicate was excluded from further analysis. Consistent with the hitchhiking model, TDP-43 was quantified within both control and mutant KIF1A co-immunoprecipitations. A total of 3138 proteins were identified in the control KIF1A bait co-immunoprecipitates (Figure 5A and Supplemental Table S2), Relative abundance of TDP-43 was above the 50th percentile of all proteins quantified using AP-MS (Figure 5B and Supplemental Table S2). Protein quantification was scored on the basis of TDP-43 peptides quantified, which did not include peptides beginning after G294 because of instrumental limitations and amino acid sequencing. TDP-43 peptide quantifications included two peptides from the N-terminal domain and nuclear localization sequence regions, two from RNA-recognition motif 1, one from the salt bridge, and two from the beginning of the C-terminal domain (Figure 5C). There was no significant difference in TDP-43 protein levels found in control and KIF1A^mut^ co-immunoprecipitate lysates (Figure 5D). However, peptide correlation analysis performed on this data set revealed that the TDP-43 peptide comprising most of the nuclear localization sequence, KMDETDASSAVK (AA), is significantly down-regulated in the KIF1A^mut^ co-immunoprecipitates compared with all other peptides detected in both KIF1A wild-type and KIF1A^mut^ precipitate (Figure 5E). These data suggest that KIF1A^mut^ shows decreased ability to associate with the nuclear localization sequence of TDP-43. There is a set of integral kinesin-cargo adaptors that modulate kinesin activity called kinesin light chain proteins (KLCs). In the KIF1A bait co-immunoprecipitates, there were no changes in KLC1 and KLC4. However, a significant decrease in KLC2 protein levels were observed in mutant KIF1A co-immunoprecipitates relative to controls (Figure 5F). The reduced association of mutant KIF1A with both TDP-43 and KLC2 may further compromise kinesin-dependent transport, which is critical for neuron structure and function.

**Figure 6:**
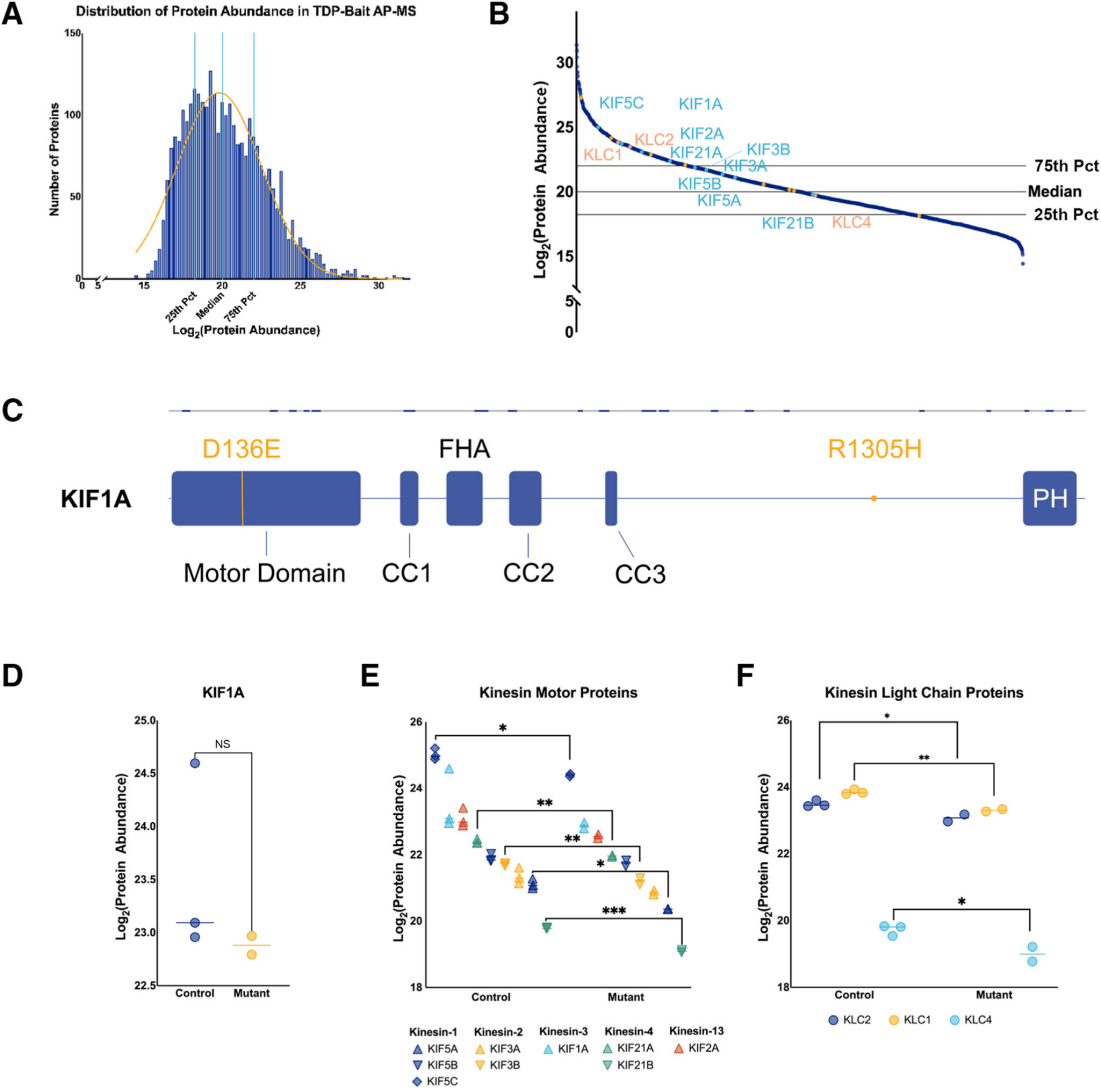
TDP-43 associated with mutant KIF1A but has decreased relationship with kinesin light chain proteins (KLCs). Neuronal protein lysates were co-immunoprecipitated from control and mutant neurons using an N-terminal TDP-43 antibody as bait. Proteins were identified and quantified using affinity-purification mass spectrometry (AP-MS). **A:** Proteins quantified in the control TDP-43 co-immunoprecipitates exhibited a gaussian distribution. **B:** Quantified proteins were sorted by protein abundance level in control neurons with kinesin motor proteins (cyan) identified as having protein abundance level above or near the median. KIF1A is above the 75th percentile (Pct). Only KIF21B is below the median. Kinesin light chain proteins, KLC1 and KLC2, locate above the 75th percentile, whereas KLC4 located slightly below the 25th percentile. **C:** Illustrated representation of KIF1A proteins and concordant domains. KIF1A mutations D136E and R1305H are denoted by gold marks in the protein. The bar above represents the regions of the protein with peptides undetected (gray) and those identified (blue). **D:** KIF1A protein levels are not significantly different between control and mutant co-immunoprecipitations for TDP-43 (unpaired two-tailed *t*-test, control mean = 23.55, mutant mean = 22.88). **E:** Other kinesin motor proteins are significantly decreased in mutant co-immunoprecipitates: KIF5C (blue diamond; control mean = 25.03, mutant mean = 24.39, unpaired two-tailed *t*-test), KIF21A (green up-triangle; control mean = 22.39, mutant mean = 21.96, unpaired two-tailed *t*-test), KIF3B (gold down-triangle; control mean = 21.72, mutant mean = 21.20), KIF5A (blue up-triangle; control mean = 21.12, mutant mean = 20.36, unpaired two-tailed *t*-test), and KIF21B (green down-triangle; control mean = 19.78, mutant mean = 19.09, unpaired two-tailed *t*-test). **F:** KLC1 (gold; control mean = 23.86, mutant mean = 23.30, unpaired two-tailed *t*-test), KLC2 (blue; control mean = 23.50, mutant mean = 23.08, unpaired two-tailed *t*-test), and KLC4 (cyan; control mean = 19.73, mutant mean = 19.01, unpaired two-tailed *t*-test) protein levels were all significantly decreased in TDP-43 co-immunoprecipitates of mutant neurons relative to control neurons. ∗*P* < 0.05, ∗∗*P* < 0.01, and ∗∗∗*P* < 0.001. CC, coiled-coiled domain; FHA, forkhead association domain; NS, nonsignificant; PH, pleckstrin homology domain.

The relationship between KIF1A and TDP-43 was further investigated using a TDP-43 bait-antibody. In this AP-MS experiment, 3061 proteins in the control and mutant co-immunoprecipitates were quantified. The control TDP-43 bait co-immunoprecipitate exhibited a normal gaussian distribution, with more than half of all proteins quantified falling the below the 50^th^ percentile (Figure 6A and Supplemental Table S3). The data showing that KIF1A was quantified at protein levels above the 75th percentile in both control and mutant co-immunoprecipitates further corroborate that KIF1A and TDP-43 interact (Figure 6B and Supplemental Table S3). A total of 52 KIF1A peptides, covering the motor domain, all three coiled-coiled domains, the forkhead association domain, and the tail domain, including the Pleckstrin homology domain, were quantified (Figure 6C). KIF1A protein levels were not differentially significant between TDP-43 bait co-immunoprecipitation AP-MS experiments (Figure 6D). Furthermore, no KIF1A peptides were significantly different between control and mutant co-immunoprecipitates. Notably, no KIF1A-mutated peptides were detected in this AP-MS experiment.

Interestingly, the TDP-43 bait co-immunoprecipitation did yield several other kinesin motor proteins, in addition to KIF1A, belonging to kinesin subfamilies: kinesin-1, kinesin-2, kinesin-4, and kinesin-13. Unexpectedly, KIF5C had the greatest associative strength with TDP-43, which was significantly decreased in the KIF1A mutant neurons relative to control neurons (Figure 6E). Similarly, the protein levels of KIF3B, KIF5A, KIF21A, and KIF21B were all significantly decreased in the TDP-43 bait immunoprecipitates from KIF1A mutant expressing cells relative to controls (Figure 6E). Finally, the association of TDP-43 with kinesin light-chain proteins and annexin protein family members was evaluated. In the TDP-43 bait immunoprecipitates, there were significant decreases in KLC1, KLC2, and KLC4 protein levels (Figure 6F). Although annexin-11 has been shown to interact with TDP-43 as a molecular tether to KIF1A-bound vesicles,^22^ annexin-11 association with TDP-43 did not differ between control and mutant KIF1A neurons. Instead, among all annexin protein family members, TDP-43 association with ANXA3 [log_2_(mutant/control) = 0.2949, *P* = 0.0424, q = 0.3099] and ANXA5 [log_2_(mutant/control) = 0.3371, *P* = 0.0301, q = 0.2741] was significantly and modestly increased in mutant KIF1A neurons compared with control neurons.

**Figure 7:**
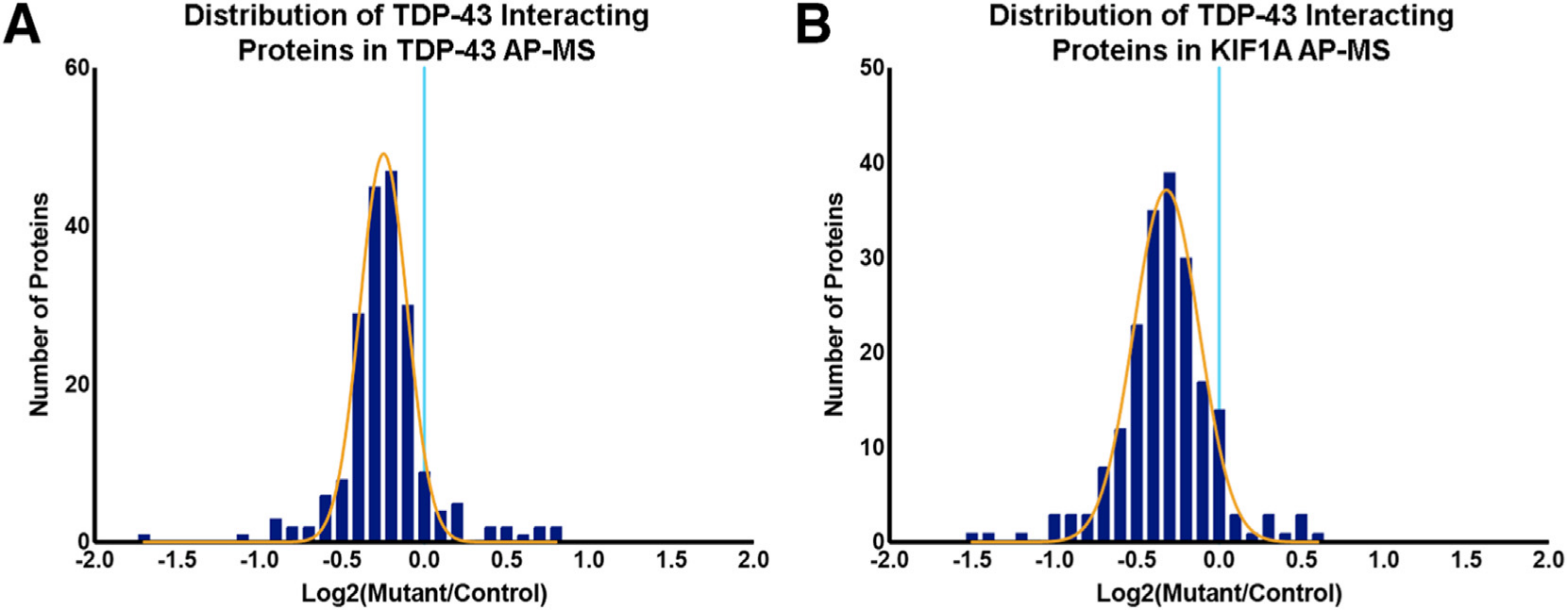
TDP-43 interacting protein associations are decreased in mutant KIF1A neurons. Frequency distributions of known TDP-43 interactor proteins quantified using TDP-43 affinity-purification mass spectrometry (AP-MS) and KIF1A AP-MS data sets filtered by the data set of Freibaum et al^23^ (2011) to evaluate the association between TDP-43 and KIF1A and known TDP-43 interactor proteins in mutant and control KIF1A neurons. Distributions are represented by bar-graph histograms (blue bars) and normal gaussian distributions (**gold curve**), and log_2_ fold change of 0 is noted by a **cyan line**. **A:** A total of 202 (of 262) TDP-43 interactor proteins were quantified in mutant and control KIF1A neurons via TDP-43 AP-MS. Proteins were binned by log_2_ fold change (mutant relative to control) at bin widths of 0.1, ranging from a minimum of –1.462 to a maximum of 0.643. The average fold change of TDP-43 interactor proteins with TDP-43 from mutant KIF1A neurons was significantly decreased relative to control KIF1A neurons (mean = –0.3275, SD = 0.2924, *P* < 0.0001; one sample *t*-test). **B:** Frequency distribution of known TDP-43 interactor proteins quantified using KIF1A AP-MS. The KIF1A AP-MS data set evaluated the association between KIF1A and known TDP-43 interactor proteins in mutant and control KIF1A neurons. A total of 201 (of 262) TDP-43 interactor proteins were quantified in mutant and control KIF1A neurons. Proteins were binned by log_2_ fold change (mutant relative to control) at bin widths of 0.1, ranging from a minimum of –1.657 to a maximum of 0.846. The average fold change of TDP-43 interactor proteins with mutant KIF1A was significantly decreased relative to control KIF1A (mean = –0.2289, SD = 0.2900, *P* < 0.0001; one sample *t*-test).

Finally, the impact of KIF1A mutation on interactions involving known TDP-43 interacting proteins, based on the comprehensive data set of Freibaum et al,^23^ was quantified. A total of 202 (77%) of 262 TDP-43 interacting proteins were quantified in the current TDP-43 AP-MS data set, wherein 65 proteins were nominally significant. The overwhelming majority (94%, 190 of 200) of quantified TDP-43 interacting proteins showed decreased interactions with TDP-43 in the mutant KIF1A neurons relative to control neurons (Figure 7A and Supplemental Table S4). The current KIF1A AP-MS data set allowed quantification of 201 (76%) of 262 TDP-43 interacting proteins, wherein 37 proteins were nominally significant. The overwhelming majority (91%, 182 of 200) of quantified TDP-43 interacting proteins showed decreased abundance in the mutant KIF1A pulldowns (Figure 7B).

Taken together, these data establish biochemical relationships between TDP-43 and several kinesin motor proteins, including KIF1A, and kinesin light chain proteins. Moreover, in mutant KIF1A neurons, most known TDP-43 interacting proteins showed decreased association with both KIF1A and TDP-43.

### KIF1A Mutant Neurons Exhibit Reduced Neuritic Outgrowth

**Figure 8:**
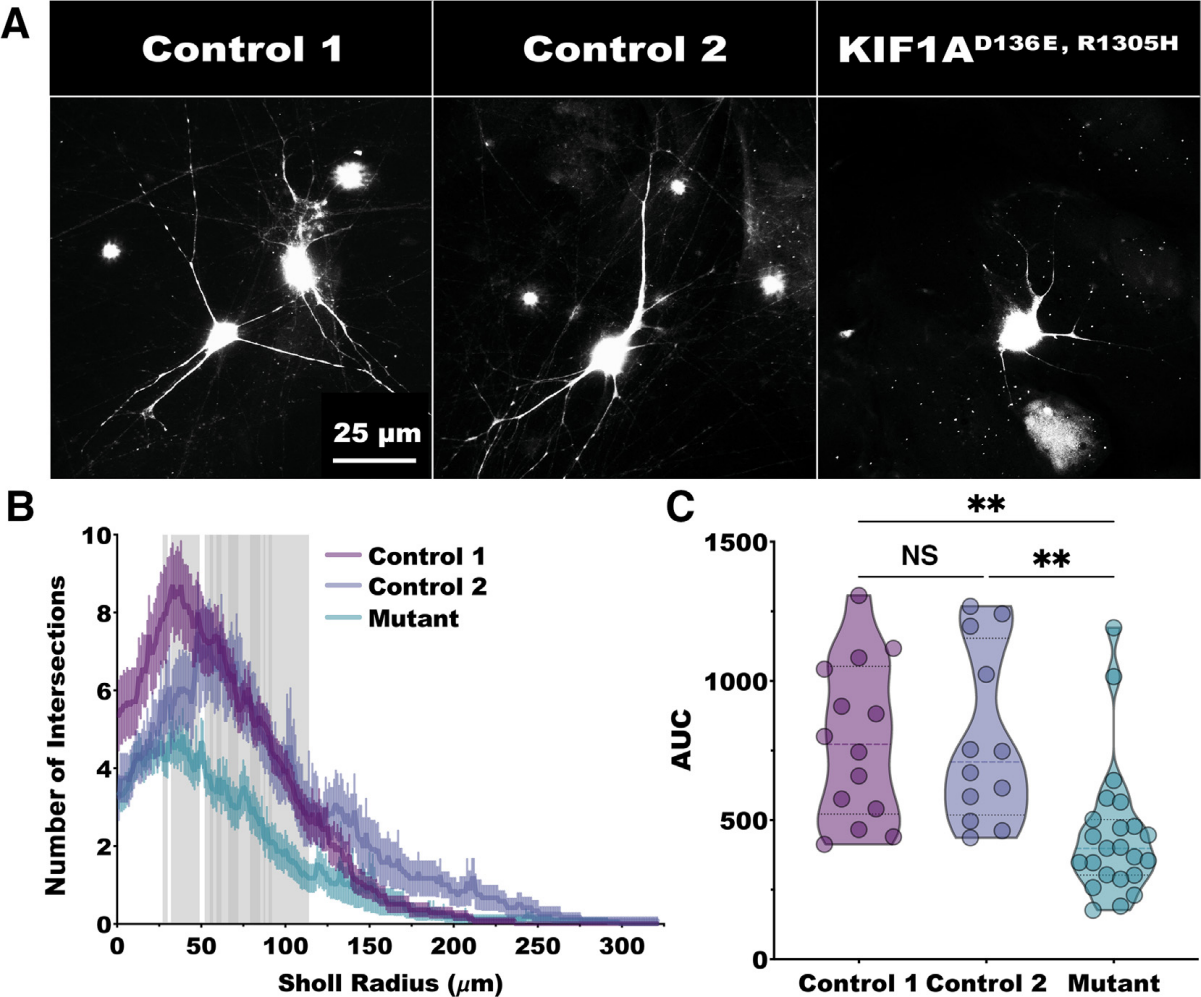
Neuronal arborization is decreased in KIF1A^D136E,^^R1305H^ induced pluripotent stem cell (iPSC) motor neurons. **A:** Representative images of iPSC-derived neurons from control 1, control 2, and KIF1A^D136^^E^^,^^R1305H^ (mutant) neurons transfected with infrared fluorescent protein 670 to highlight individual neurons. **B:** Dendritic arborization of neurons was measured via Sholl analysis for control 1 (purple), control 2 (blue), and KIF1A mutant (green). **Internal bold lines** and **external pale lines** indicate the mean and SEM, respectively, at each radius. Statistical analysis was conducted using two-way repeated measures analysis of variance (ANOVA) with Tukey multiple comparison test (F = 3.648, ε = 0.8161). Control 1 and control 2 were never significantly different in number of intersections from soma at any distance from the soma. KIF1A mutant neuron arborization was significantly reduced compared with control 1 beginning at 19 μm from the soma and reaching 114 μm (light gray). Similarly, KIF1A mutant neuron arborization was significantly reduced compared with control 2 beginning at 55 μm from the soma and reaching 92 μm (dark gray). **C:** The area under the curve (AUC; intersections by μm2) derived from Sholl analysis are shown for individual control 1 (purple), control 2 (blue), and KIF1A mutant (green) neurons. **Dashed lines** within the violin plot probability densities indicate the mean and interquartile range. Statistical analysis was completed using a one-way ANOVA with Bonferroni multiple comparison test (F = 9.576). Control 1 (mean = 784.0) and control 2 (mean = 791.0) AUCs were not significantly different. Mutant (mean = 447.5) AUC was significantly decreased compared with control 1 and control 2. *n* = 14 for control 1; *n* = 12 for control 2; *n* = 23 for KIF1A mutant neurons. ∗∗*P* < 0.01. Scale bars = 25 μm (**A**). NS, nonsignificant.

The changes to the binding capabilities of mutant KIF1A may have downstream consequences related to protein trafficking and the growth and maintenance of the synaptic architecture. Loss of morphologic complexity represents a structural hallmark of multiple neurodegenerative diseases.^24^^,^^25^ To determine if KIF1A^mut^ motor neurons exhibit altered morphologic complexity, mature neurons were transduced with infrared fluorescent protein 670 to facilitate whole neuron tracing (Figure 8A). Sholl analysis indicates that the dendritic arborization of KIF1A^mut^ motor neurons was significantly reduced in the 25- to 115-μm range, compared with control motor neurons (Figure 8B). The area under the curve of the Sholl analysis profile for individual KIF1A^mut^ neurons was also significantly reduced compared with KIF1A^WT^ neurons (Figure 8C). These data indicate that these KIF1A mutations elicit pathogenic effects on neuronal architecture as a marker of neurodegeneration.

## Discussion

KIF1A^mut^ iPSCs were generated from fibroblasts donated by a patient presenting with an asymmetric syndrome of spasticity and clonus followed by parkinsonism with dystonia. The evolving parkinsonism and lack of corticospinal tract involvement on magnetic resonance tractography made primary lateral sclerosis unlikely. Some aspects, such as marked asymmetry, lack of response to lower doses of levodopa, presence of myoclonus, early fixed dystonia, and positive DATSCAN, raised the possibility of corticobasal syndrome. However, the partial responsiveness to high-dose levodopa with improvement in parkinsonism and dystonia, development of levodopa-induced dyskinesia, and absence of cognitive impairment were atypical for the classic presentation of corticobasal syndrome. On the other hand, neither the patient's clinical features nor the pathologic findings observed for his father were consistent with idiopathic Parkinson disease, hereditary spastic paraparesis, or classic motor neuron diseases. The findings on DATSCAN were consistent with neurodegenerative involvement of nigrostriatal pathways. Tractography indicated a process affecting the cerebellar-brainstem connections but sparing the corticospinal tracts. The patient's father had presented with a PSP-like clinical phenotype, whereas pathologic re-assessment of his brain tissue confirmed a tauopathy felt to be more consistent with CBD and involving extensive brainstem TDP-43 pathology, including the substantia nigra, tectum, and oculomotor nuclei of the midbrain, the basis pontis, and medullary olives. Taken together, this presentation is suggestive of an atypical parkinsonism associated with a corticobasal syndrome/PSP clinical phenotype, with diffuse tauopathy and brainstem TDP-43 pathology. This constellation of clinical symptoms and neuropathologic findings is reminiscent of a recently described clinicopathologic subtype of CBD, characterized by PSP-like clinical presentations, severe tau pathology in the olivopontocerebellar system, brainstem TDP-43 pathology, and low frequency of the *MAPT* H1 haplotype.^26^ No associations with known genetic risk factors for TDP43 pathology in *TMEM106B* or *GRN* genes were identified in that cohort, but whole-genome screening was not performed.

Genetic testing of genes associated with familial Parkinson Plus syndromes, including those with PSP or CBS phenotypes, such as *C9orf72*, *MAPT*, *TARDB*, *VCP*, and *GRN*, was unremarkable. The patient and his father carried two VUSs in the KIF1 gene, increasing the likelihood that their presentation could be related to KIF1A gene dysfunction. Mutations in KIF1A have been shown to change the protein's motility and cargo-binding dynamics. Although D136E is a conservative substitution, mutations in the motor domain near D136E are associated with a gain-of-function mechanism that increases microtubule- and/or ATP-binding and trafficking processivity.^27^, ^28^, ^29^, ^30^ Here, KIF1A has a second mutation, R1305H, of unknown significance. This mutation is situated between the third coiled-coil region and the Pleckstrin homology domain, which are essential domains for cargo interaction and binding.^27^, ^28^, ^29^, ^30^, ^31^

KIF1A mutations are associated with a spectrum of neurologic manifestations, and the current study broadens this to include atypical parkinsonism. Although KIF1A-associated neurologic disorders can affect the central nervous system in addition to peripheral nerve, reports that include pathologic studies of the brain are exceedingly rare.^32^ To our knowledge, there are no prior published studies that examine the neuropathology of aggregate-prone proteins (TDP-43, tau, α-synuclein, or Aβ) in the context of KIF1A-associated neurologic disorders. The current clinical-pathologic and patient iPSC-derived motor neuron studies support a pathologic role for these new KIF1 VUSs in processes relevant to TDP-43 biology and neuronal arborization. Significant loss of morphologic complexity, as observed here, is associated with decreased synaptic density and function^17^ and represents a major hallmark of multiple neurodegenerative diseases.^24^^,^^25^ Given the diverse clinical manifestations associated with KIF1A, it remains to be determined how different mutations may lead to selective patterns of degeneration and functional impairment that may confer risk for different clinical syndromes.

One such mechanism could involve dysregulation of TDP-43 trafficking. A major function of KIF1A in mature neurons is facilitating the transport of key cargos away from the soma throughout the axodendritic arbor.^33^^,^^34^ Transport of these cargos, including synaptic vesicles, dense core vesicles, and endolysosomes, plays critical roles in maintaining neuronal morphology and plasticity.^35^ Recent evidence suggests that KIF1A, and the KCL adaptors, may act as mediators of TDP-43 containing RNA granule trafficking into axons.^22^ Furthermore, the patient's father, who had identical mutations in KIF1A, had an atypical pattern of predominantly midbrain and brainstem cytoplasmic TDP-43 aggregation with occasional motor neuron dystrophic neurites. In contrast, increased nuclear retention of TDP-43 was observed, which may reflect decreased distal trafficking of TDP-43. Notably, both the iPSC-neuron shift favoring nuclear to cytoplasmic TDP-43 distribution and the pathologic cytoplasmic/neuritic aggregation of TDP-43 would act to diminish normal TDP-43 function in the axon. Additionally, a diminished interaction between the nuclear localization sequence of TDP-43 and KIF1A^mut^ may play a yet unknown role in regulating extranuclear TDP-43 function.

The contrasting patterns of TDP-43 alterations in the patient's father's brainstem and the patient's iPSC neurons could be due to differences in specific neuron type or stage of disease. Nuclear retention of soluble TDP-43 in KIF1A^mut^ iPSC neurons may represent an early pathogenic event, with cytosolic and neuritic aggregations occurring in later stages. Indeed, given the aberrant interaction between KIF1A^mut^, its adapters, and TDP-43 identified from these iPSC-neuron proteomic studies, the loss of transport complex integrity may result in TDP-43 cytoplasmic accumulation over time, and this will be an area for future study. Furthermore, post-mortem analysis often favors aggregated over soluble proteins because of residual enzymatic activities before fixation. In either case, the clinical, pathologic, and experimental data all implicate these particular KIF1A VUSs in age-related neurodegeneration, rather than the more common childhood or young adult presentation.

A limitation of the current study is that the potential impact of the KIF1A mutation on the pathobiology of tau, the other aggregated protein observed in the father's brain, could not be assessed. Immunocytochemistry for phosphorylated tau was performed on KIF1A^mut^ and KIF1A control neurons. However, we were unable to identify phosphorylated tau pathology in KIF1A^mut^ neurons (data not shown). These negative data are likely a result of the relatively immature developmental stage of iPSC-derived neurons, in which the fetal isoform^36^ is predominantly expressed. In iPSC neurons, expression of four repeat isoforms of tau implicated in CBD/PSP pathology is more than two orders of magnitude less than fetal isoforms.^37^^,^^38^ Although the potential role of this KIF1A mutation on tau biology remains unknown, kinesins, in general, are reported to interact with tau.^39^

In summary, we report the unusual clinical phenotype associated with KIF1A^D136E,R1305H^ mutations in a father-son pair, which is associated with atypical parkinsonism in the son, and a CBD-like pattern of tau pathology with an unusual pattern of brainstem-predominant TDP-43 pathology in the father. Motor neurons differentiated from patient-derived iPSC lines exhibit altered TDP-43 subcellular distribution, a decrease in KIF1A–TDP-43 biochemical associations, and simplified dendritic arbors. This report broadens the clinical spectrum of KIF1A-associated neurologic disease with a new variant linked to atypical parkinsonism, tau and TDP-43 pathology. Endogenous expression of these VUSs in KIF1A disrupts TDP-43 cellular interactions in human iPSC motor neuron cultures and elicit neuronal architectural deficits similar to those observed in neurodegenerative diseases.

## Disclosure Statement

None declared.

## Supplemental Data

Supplemental Figure S1 Characterization of KIF1A^D136E, R1305H^ induced pluripotent stem cell (iPSC) lines. **A:** Karyotyping was performed to evaluate for chromosomal abnormalities. No abnormalities were detected. Twenty cells were counted, eight analyzed, and four karyogrammed at a band resolution of 400 ± 25 nm. **B:** Immunofluorescence assessment of pluripotency markers stage-specific embryonic antigen-1 (SSEA1), tumor-related antigen-1-81 (TRA-1-81), and octamer-binding transcription factor 4A (OCT4a) in KIF1A^mut^ iPSCs. Pluripotency of KIF1A^mut^ iPSCs was confirmed using all three markers. **C:** Expression of germ layer gene markers paired box 6 (PAX6), SRY-box transcription factor 1 (SOX1), SOX17, C-X-C motif chemokine receptor 4 (CXCR4), forkhead box protein F1 (FOXF1), heart and neural crest derivatives expressed 1 (HAND1) was assessed in iPSCs following trilineage differentiation. Differentiated cells (blue) show up-regulation of transcript levels relative to undifferentiated cells (yellow). Statistical analysis was conducted using two-tailed unpaired *t*-test. **D:** Sanger sequencing shows iPSCs harbor both wild-type and mutant alleles. **Arrows** indicate genomic mutation; yellow highlights the codon. ∗*P* < 0.05, ∗∗*P* < 0.01, ∗∗∗*P* < 0.001, and ∗∗∗∗*P* < 0.0001 versus undifferentiated cells. Scale bar = 40 μm (**B**).
