# Supplementary figures and images for: Novel Kinesin Family Member 1A Variants Linked to Atypical Parkinsonism Elicit Altered Neuronal Transactive Response DNA Binding Protein 43 kDa Interactions and Dendritic Atrophy
Source: Am J Pathol. 2025 Jun 19;195(11):2161–75. doi: 10.1016/j.ajpath.2025.05.018 (PMC12597689; doi:10.1016/j.ajpath.2025.05.018)

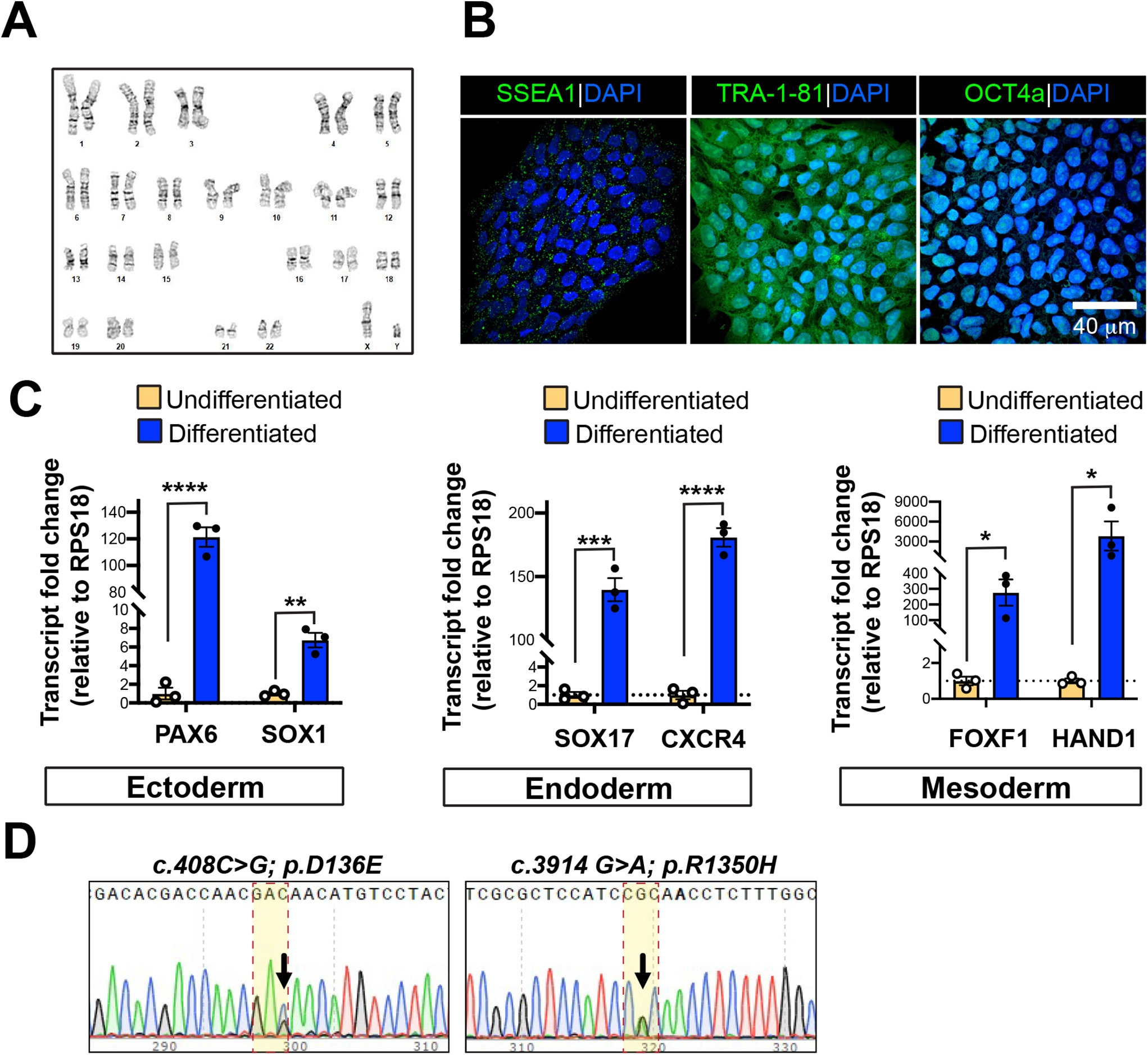

Supplement: Supplemental Figure S1 — Characterization of KIF1AD136E, R1305H induced pluripotent stem cell (iPSC) lines. A: Karyotyping was performed to evaluate for chromosomal abnormalities. No abnormalities were detected. Twenty cells were counted, eight analyzed, and four karyogrammed at a band resolution of 400 ± 25 nm. B: Immunofluorescence assessment of pluripotency markers stage-specific embryonic antigen-1 (SSEA1), tumor-related antigen-1-81 (TRA-1-81), and octamer-binding transcription factor 4A (OCT4a) in KIF1Amut iPSCs. Pluripotency of KIF1Amut iPSCs was confirmed using all three markers. C: Expression of germ layer gene markers paired box 6 (PAX6), SRY-box transcription factor 1 (SOX1), SOX17, C-X-C motif chemokine receptor 4 (CXCR4), forkhead box protein F1 (FOXF1), heart and neural crest derivatives expressed 1 (HAND1) was assessed in iPSCs following trilineage differentiation. Differentiated cells (blue) show up-regulation of transcript levels relative to undifferentiated cells (yellow). Statistical analysis was conducted using two-tailed unpaired t-test. D: Sanger sequencing shows iPSCs harbor both wild-type and mutant alleles. Arrows indicate genomic mutation; yellow highlights the codon. ∗P < 0.05, ∗∗P < 0.01, ∗∗∗P < 0.001, and ∗∗∗∗P < 0.0001 versus undifferentiated cells. Scale bar = 40 μm (B). [file figs1.jpg]
