# Supplementary material for: Novel Kinesin Family Member 1A Variants Linked to Atypical Parkinsonism Elicit Altered Neuronal Transactive Response DNA Binding Protein 43 kDa Interactions and Dendritic Atrophy
Source: Am J Pathol. 2025 Jun 19;195(11):2161–75. doi: 10.1016/j.ajpath.2025.05.018 (PMC12597689; doi:10.1016/j.ajpath.2025.05.018)
Supplement: Supplemental Table S1 [file mmc1.docx]

Supplemental Table S1. Patient iPSC Lines.

| **Line ID** | **Figure ID** | **Genotype** | **Sex** | **Source** |
| --- | --- | --- | --- | --- |
| JH082 | Control 1 | Healthy Control | Male | Johns Hopkins |
| CS15iCTR | Control 2 | Healthy Control | Male | Cedars Sinai iPSC Core |
| CS03iCTR | Control 3 | Healthy Control | Male | Cedars Sinai iPSC Core |
| Pitt27 | Mutant | KIF1A^D136E, R1305H^ | Male | University of Pittsburgh |
