# Supplementary material for: Novel Kinesin Family Member 1A Variants Linked to Atypical Parkinsonism Elicit Altered Neuronal Transactive Response DNA Binding Protein 43 kDa Interactions and Dendritic Atrophy
Source: Am J Pathol. 2025 Jun 19;195(11):2161–75. doi: 10.1016/j.ajpath.2025.05.018 (PMC12597689; doi:10.1016/j.ajpath.2025.05.018)
Supplement: Supplemental Table S4 [file mmc4.docx]

Supplemental Table S4. Distribution of TDP-43 Interacting Proteins Quantified Using TDP-43 AP-MS.

| **Log_2_Mut/Ctrl**  **Bin Range** | | **Quantified Proteins** | |
| --- | --- | --- | --- |
|  | |  | |
| **Min** | **Max** | **Number** | **Percentage** |
| -1.5 | -1.4 | 1 | 0.5% |
| -1.4 | -1.3 | 1 | 0.5% |
| -1.3 | -1.2 | 0 | 0.0% |
| -1.2 | -1.1 | 1 | 0.5% |
| -1.1 | -1.0 | 0 | 0.0% |
| -1.0 | -0.9 | 3 | 1.5% |
| -0.9 | -0.8 | 3 | 1.5% |
| -0.8 | -0.7 | 8 | 4.0% |
| -0.7 | -0.6 | 12 | 6.0% |
| -0.6 | -0.5 | 23 | 11.4% |
| -0.5 | -0.4 | 35 | 17.3% |
| -0.4 | -0.3 | 39 | 19.3% |
| -0.3 | -0.2 | 30 | 14.9% |
| -0.2 | -0.1 | 17 | 8.4% |
| -0.1 | 0.0 | 14 | 6.9% |
| 0.0 | 0.1 | 3 | 1.5% |
| 0.1 | 0.2 | 1 | 0.5% |
| 0.2 | 0.3 | 3 | 1.5% |
| 0.3 | 0.4 | 1 | 0.5% |
| 0.4 | 0.5 | 3 | 1.5% |
| 0.5 | 0.6 | 1 | 0.5% |
